# Frequent alcohol drinking is associated with lower prevalence of self-reported common cold: a retrospective study

**DOI:** 10.1186/1471-2458-12-987

**Published:** 2012-11-16

**Authors:** Eriko Ouchi, Kaijun Niu, Yoritoshi Kobayashi, Lei Guan, Haruki Momma, Hui Guo, Masahiko Chujo, Atsushi Otomo, Yufei Cui, Ryoichi Nagatomi

**Affiliations:** 1 Department of Medicine & Science in Sports & Exercise, Tohoku University Graduate School of Medicine, Sendai, 980-8575, Japan; 2 Division of Biomedical Engineering for Health & Welfare, Tohoku University Graduate School of Biomedical Engineering, Sendai, 980-8575, Japan; 3 Lab of Nutritional Epidemiology, Department of Nutrition and Food Hygiene, School of Public Health, Tianjin Medical University, Tianjin, 300070, People’s Republic of China

**Keywords:** Alcohol, Drinking, Dietary history, Common cold

## Abstract

**Background:**

Alcohol intake has been associated with reduced incidence of common cold symptoms in 2 European studies. However, no study has addressed the association between the frequency of alcohol intake and the incidence of common cold. This study aimed to investigate the association between the amount and frequency of alcohol drinking and the retrospective prevalence of common cold in Japanese men.

**Methods:**

This retrospective study included men who participated in an annual health examination conducted in Sendai, Japan. The frequency of common cold episodes in the previous year was self-reported. The weekly frequency and amount of alcohol consumed, as well as the type of alcoholic drink, were reported by a brief-type self-administered diet history questionnaire. Logistic regression models were used to analyze the association between the amount and frequency of alcohol intake and the retrospective prevalence of common cold.

**Results:**

Among 899 men, 83.4% of the subjects reported drinking alcohol, and 55.4% of the subjects reported having experienced at least one episode of common cold in the previous year. Compared with non-drinkers, the adjusted odds ratios (ORs) with 95% confidence intervals (CIs) for having had 1 or more episodes of common cold during the past year across categories of alcohol intake frequency of 3 or less, 4–6, and 7 days/week were 0.827 (0.541–1.266), 0.703 (0.439–1.124), and 0.621 (0.400–0.965), respectively (*P* for trend = 0.025); the adjusted ORs with 95% CIs for having had of 2 or more episodes of common cold across the same categories were 0.642 (0.395–1.045), 0.557 (0.319–0.973), and 0.461 (0.270–0.787), respectively (*P* for trend = 0.006). Compared with subjects who consumed 11.5–35.8 g of alcohol per day, the non-drinkers were significantly more likely to experience 2 or more episodes of common cold (OR, 1.843; 95% CI, 1.115–3.047).

**Conclusion:**

The frequency, not the amount, of alcohol intake was significantly related to lower prevalence of self-reported common cold episodes in Japanese men.

## Background

A common cold is one of the most frequently occurring illnesses in humans. According to a survey conducted in the United States (U.S.) [1], the estimated average number of common cold episodes per adult per year was 2.5. The common cold is not harmful in most cases, and people recover within a few days; however, those who suffer from chronic respiratory diseases, such as asthma [2] or chronic obstructive pulmonary disease (COPD) [3], have difficulties controlling their symptoms after catching a cold. Furthermore, common cold causes huge economic burden. It has been estimated that the direct cost of common cold amounts to approximately $17 billion per year [1]. In addition, respiratory symptoms are the major causes for sickness absence among workers [4], and common cold impacts on working productivity, resulting in average of 8.7 lost work hours per each common cold experienced by a working adult [5]. Therefore, it is important to seek for effective measures or life style that may potentially reduce common cold incidence, as well as to seek for potential risk factors.

According to an experimental study by Cohen et al., alcohol intake contributed to the reduction in the risk of a common cold among non-smokers [6]. Takkouche et al. surveyed the relationship between the incidence of common cold and alcohol intake in a prospective cohort study and concluded that as the amount of wine intake, not total alcohol, increased, the relative risk of having a common cold significantly decreased [7]. It is, however, unclear whether alcohol or other components in wine had a preventive effect on common cold.

The amount and types of alcohol intake vary in different countries. The average amount of alcohol intake per drinker in the United Kingdom (U.K.) and Spain, where the above mentioned 2 studies were conducted, is more than 1.5 times higher than that in Japan [8]. In addition, in these countries, wine and beer are the most frequently consumed beverage types [8]. On the other hand, the Japanese consume mainly beer and Japanese spirits [8,9]. If alcohol *per se* has a potential role in reducing common cold incidence, the risk reduction may not depend upon the type of alcoholic drinks but on the amount or the frequency of alcohol intake.

To date, no study has addressed the association between the prevalence of common cold and alcohol intake in the Japanese population. Therefore, the aim of this research was to investigate whether there was a correlation between the amount and frequency of alcohol intake and the retrospective prevalence of self-reported common cold in Japanese men.

## Methods

### Study design

The study was based on data from the Oroshisho longitudinal study (from August 2008 to August 2011) composed of a dynamic cohort of adult employees working at the Sendai Oroshisho Center, which is one group (over 120 enterprises) of small and medium enterprises in Sendai, northern Japan. The survey included the results of annual health examinations and additional questionnaires and assessments. The details of the study have been described previously [10-13]. Data from the second year of the cohort study were used for this analysis, because the information on common cold was not collected at baseline.

### Subjects

A total of 1263 men and women received health examinations. Of them, 1215 agreed to participate in this survey and provided written consent forms for their data to be analyzed (response rate = 96.2%). However, because of significant gender differences in the amount and frequency of alcohol intake (p < 0.001) and the insufficient number of female participants for logistic analysis, females (*n* = 282) were excluded from the final analysis. Males with missing data were also excluded (*n* = 34), resulting in a total of 899 subjects for the present analyses. The study protocol was approved by the Institutional Review Board of the Tohoku University Graduate School of Medicine.

### Common cold

Participants were asked how many times they had common cold in the previous year, and to select one of the following three options; 1) none, 2) once, and 3) twice or more. History of influenza was excluded from the definition of common cold. Definitions of common cold for analyses were whether or not participants experienced 1) 1 or more episodes of common cold and 2) 2 or more episodes of common cold.

### Alcohol intake

The frequency of alcohol intake per week was estimated based on the data collected from a brief-type self-administered diet history questionnaire (BDHQ). The BDHQ assesses the dietary habits of the previous month, and the participants are required to provide the average frequency of each food intake per week [14]. For assessment of alcohol intake, the participants checked 1 of the following 9 frequency categories: never, less than once, and ranging from “1 day per week” to “7 days per week.” For analyses, drinking frequency was categorized into 4 groups: 0, ≤3, 4–6, and 7 days/week. In addition, average numbers of the following types of 5 alcoholic beverages per session were considered: Japanese sake, beer, Japanese spirits, spirits, and wine. From these data, the average estimated amount of pure alcohol intake per day was calculated [15]. Then, the amount of alcohol was categorized into non-drinkers and tertiles.

### Assessment of other variables

Information on age, sex, occupation, educational level, and smoking status was collected using questionnaires in addition to conventional annual health examinations. Based on occupation, a participant was categorized as an office worker or non-office worker. Office workers are employees who work in an office, especially those engaged in clerical or administrative work. Non-office workers include those working as salesmen, or those engaged in jobs that require physical labors such as in manufacture or in carrier business. Educational level was categorized on the basis of the duration of education: greater than 12 years or less. The participants chose their smoking status from non-smoker, ex-smoker, and current smoker. Metabolic syndrome (MetS) was defined as the presence of 3 or more of the following 5 risk components: 1) waist circumstance modified for Japanese as ≥85 cm, 2) average casual blood pressure ≥130/85 mmHg and/or currently taking anti-hypertensive medication, 3) triglycerides ≥150 mg/dL and/or currently taking lipid-lowering medication, 4) high-density lipoprotein cholesterol <40mg/dL and/or currently taking drug treatment for reduced HDL-cholesterol, and 5) fasting glucose ≥100 mg/dL and/or currently taking oral anti-diabetic medication [16]. Body mass index (BMI) was calculated as weight/height^2^ (kg/m^2^). Average sleep duration per day was categorized into 3 groups: <7, 7–8, and ≥8 hours. Weekly physical activity (PA) level was calculated using the International Physical Activity Questionnaire (IPAQ) [17]. PA level was determined as daily activity of less or more than 10 minutes. Daily intakes of calories (kcal/day), vitamin C (mg/day), and zinc (mg/day) were calculated from the BDHQ and were divided into quartiles. The Japanese version of the Self-Rating Depression Scale (SDS) was used to assess depressive symptoms [18]. For analyses, having depressive symptoms was defined as score 45 or more out of 80 points.

### Statistical analysis

In this study, because of the distribution of age and BMI was not normal, the common logarithm was applied to normalize the data before statistical analysis. Logistic regression for categorical variables and analysis of variance for continuous variables were used to assess the difference in basic characteristics according to the categorized amount and frequency of alcohol intake. Logistic regression models were also used to analyze the association between the amount and frequency of alcohol intake and the retrospective prevalence of common cold. The odds ratios (ORs) were adjusted with the following potential confounding factors: age, education, occupation, smoking status [19], BMI, PA [20], depressive symptoms [21,22], sleep duration [23], MetS, calorie intake, vitamin C intake [24], and zinc intake [25]. For all statistical analyses, the level of significance was determined at *P* < 0.05. Statistical Package for the Social Sciences (SPSS) software 18.0 (SPSS Japan Inc., Tokyo, Japan) was used for all analyses.

## Results

### Basic characteristics

In our study population, 55.4% and 19.9% of the subjects reported having experienced common cold episodes at least once and twice or more during the previous year, respectively. The average amount of alcohol intake was 30.0 g/day with standard deviation of 29.1. Distribution of alcoholic beverages by type was as follows: 12.3% for Japanese sake; 42.9%, beer; 30.6%, Japanese spirits; 8.1%, spirits; and 6.1%, wine. Of the subjects, 16.6% were non-drinkers, and the percentage of those who drank 1 or 2 or more types of alcoholic beverages were 28.9% and 54.5%, respectively. Intake of both beer and Japanese spirits was observed in 63.5% of subjects who drank 2 or more types of alcoholic beverages. The frequency and amount of alcohol intake was significantly positively correlated (*r* = 0.698, p < 0.001).

The basic characteristics of the participants according to the categorized frequency and amount of alcohol intake are shown in Table 1 and Table 2, respectively. Compared to the subjects who did not drink, those who consumed alcohol every day or more than 35.8 g/day were more likely to be older, currently smoking, have lower BMI, consume more calories, and sleep 7–8 hours per day. They were less likely to never have smoked, consume less calories, and sleep less than 7 hours. No significant relationship was found between the amount and frequency of alcohol intake and other variables.

**Table 1:** Basic characteristics of the study subjects according to the categorized frequency of alcohol intake

	**Frequency of alcohol intake (day/week)**				***P *for trend**^**a**^
	**0**	**≤3**	**4-6**	**7**	
	**(n = 149)**	**(n = 294)**	**(n = 169)**	**(n = 287)**	
Age (year)^b^	45.0 (38.0–55.0)	41.5 (36.0–52.3)	48.0 (38.0–55.0)	51.0 (42.0–57.0)	< 0.001
Education (%)					
≥12 years	35.6	59.5	53.3	38.7	0.135
Occupation (%)					
Non office work	11.4	24.1	23.1	20.2	0.234
Smoking status (%)					
Never smoker	39.6	39.1	40.2	27.2	0.003
Ex smoker	9.4	15.6	11.2	12.2	0.976
Current smoker	51.0	45.2	48.5	60.6	0.004
BMI (kg/m^2^)^b^	23.8 (21.6–26.1)	23.8 (21.7–26.0)	23.6 (22.1–25.5)	22.8 (20.9–25.0)	< 0.001
Physical activity (%)					
≥10min/day	71.1	81.6	78.7	80.1	0.166
Depressive symptoms (%)					
SDS ≥45	36.9	28.2	29.6	32.4	0.759
Sleep duration (%)					
<7 hour	53.7	54.4	53.3	37.3	< 0.001
7-8 hour	32.2	33.3	33.7	44.3	0.005
≥8 hour	14.1	12.2	13.0	18.5	0.089
Metabolic syndrome (%)					
Yes	30.9	32.3	30.2	27.2	0.247
Calorie intake (%)					
1st (≤1519.0kcal)	31.5	28.9	23.7	18.5	0.001
2nd (1519.0-1877.0kcal)	26.8	28.2	21.3	23.0	0.146
3rd (1877.0-2296.9kcal)	22.8	23.1	21.9	29.6	0.078
4th (>2296.9kcal)	18.8	19.7	33.1	28.9	0.001
Vitamin C intake (%)					
1st (≤56.0mg/d)	24.2	22.1	24.3	28.6	0.139
2nd (56.0-82.0mg/d)	24.2	23.5	24.3	27.5	0.313
3rd (82.0-121.5mg/d)	31.5	25.2	26.0	20.9	0.026
4th (>121.5mg/d)	20.1	29.3	25.4	23.0	0.796
Zinc intake (%)					
1st (≤5.8mg/d)	24.8	22.8	23.1	28.2	0.259
2nd (5.8-7.3mg/d)	22.8	28.2	23.7	23.3	0.572
3rd (7.3-9.2mg/d)	24.2	24.8	24.9	26.1	0.639
4th (>9.2mg/d)	28.2	24.1	28.4	22.3	0.302

^a^ Analysis of variance for continuous variables or logistic regression for categorical variable.

^b^ Medians and interquartile range in parentheses.

**Table 2:** Basic characteristics of the study subjects according to the categorized amount of alcohol intake

	**Amount of alcohol intake (g/day)**^**a**^				***P *for trend**^**b**^
	**Non-drinking**	**≤11.5**	**11.5-35.8**	**>35.8**	
	**(n = 149)**	**(n = 250)**	**(n = 249)**	**(n = 251)**	
Age (years)^c^	45.0 (38.0–55.0)	42.0 (36.0–53.0)	47.0 (38.0–56.0)	50.0 (42.0–57.0)	< 0.001
Education (%)					
≥12 years	35.6	56.8	49.0	44.6	0.717
Occupation (%)					
Non office work	11.4	25.6	21.3	20.3	0.270
Smoking status (%)					
Never smoker	39.6	41.6	36.1	26.7	0.001
Ex smoker	9.4	16.0	12.4	11.6	0.899
Current smoker	51.0	42.4	51.4	61.8	0.001
BMI (kg/m^2^)^c^	23.8 (21.6–26.1)	23.7 (21.9–25.9)	23.3 (21.5–25.5)	23.1 (21.1–25.3)	0.009
Physical activity (%)					
≥10 min/day	71.1	83.6	76.7	80.9	0.209
Depressive symptoms (%)					
SDS ≥45	36.9	27.2	29.7	33.5	0.937
Sleep duration (%)					
<7 hour	53.7	52.8	47.8	42.2	0.009
7-8 hour	32.2	33.2	39.4	40.2	0.042
≥8 hour	14.1	14.0	12.9	17.5	0.343
Metabolic syndrome (%)					
Yes	30.9	28.0	32.9	28.7	0.956
Calorie intake (%)					
1st (≤1519.0kcal)	31.5	33.6	26.1	11.6	< 0.001
2nd (1519.0-1877.0kcal)	26.8	28.8	23.3	21.9	0.098
3rd (1877.0-2296.9kcal)	22.8	20.8	26.9	28.3	0.068
4th (>2296.9kcal)	18.8	16.8	23.7	38.2	< 0.001
Vitamin C intake (%)					
1st (≤56.0mg/d)	24.2	26.8	22.5	25.9	1.000
2nd (56.0-82.0mg/d)	24.2	24.8	24.9	25.9	0.697
3rd (82.0-121.5mg/d)	31.5	22.4	25.3	23.5	0.224
4th (>121.5mg/d)	20.1	26.0	27.3	24.7	0.408
Zinc intake (%)					
1st (≤5.8mg/d)	24.8	25.2	25.3	24.3	0.884
2nd (5.8-7.3mg/d)	22.8	28.0	23.7	24.3	0.826
3rd (7.3-9.2mg/d)	24.2	24.8	26.5	24.7	0.846
4th (>9.2mg/d)	28.2	22.0	24.5	26.7	0.865

^a^ Drinkers were categorized into tertiles.

^b^ Analysis of variance for continuous variables or logistic regression for categorical variables.

^c^ Medians and interquartile range are presented in parentheses.

### Common cold and alcohol

Table 3 presents the association of the frequency of alcohol intake with the retrospective prevalence of common cold. Compared with non-drinkers, the crude ORs (95% confidence interval [CI]) for having had 1 or more episodes of common cold in the previous year across categories of alcohol intake frequency of ≤3, 4–6, and 7 days per week were 0.964 (0.644–1.444), 0.744 (0.476–1.163), and 0.599 (0.400–0.895), respectively (*P* for trend = 0.002). The association remained present after controlling for potential confounders (*P* for trend = 0.025). In addition, compared with non-drinkers, the crude ORs (95% CI) for having had 2 or more episodes of common cold in the previous year across the aforementioned categories of alcohol intake frequency were 0.813 (0.513–1.289), 0.682 (0.401–1.162), and 0.543 (0.334–0.884), respectively (*P* for trend = 0.009). Adjustment for potential confounders resulted in a clearer association, and the adjusted ORs (95% CI) were 0.642 (0.395–1.045), 0.557 (0.319–0.973), and 0.461 (0.270–0.787), respectively (*P* for trend = 0.006).

**Table 3:** Odds ratios for having had common cold in the previous year across alcohol intake frequency

	**Frequency of alcohol intake (day/week)**				***P *for trend**^**a**^
	**0**	**≤3**	**4-6**	**7**	
≥1 common cold^b^ (cases/subjects)	91/149	177/294	91/169	139/287	
Crude OR(95% CI)	1	0.964 (0.644–1.444)	0.744 (0.476–1.163)	0.599 (0.400–0.895)^d^	0.002
Adjusted OR^c^ (95% CI)	1	0.827 (0.541–1.266)	0.703 (0.439–1.124)	0.621 (0.400–0.965) ^d^	0.025
≥2 common cold^b^ (cases/subjects)	38/149	64/294	32/169	45/287	
Crude OR (95% CI)	1	0.813 (0.513–1.289)	0.682 (0.401–1.162)	0.543 (0.334–0.884) ^d^	0.009
Adjusted OR^c^ (95% CI)	1	0.642 (0.395–1.045)	0.557 (0.319–0.973) ^d^	0.461 (0.270–0.787) ^d^	0.006

^a^ Calculated by using a logistic regression model.

^b^ Frequency of self-reported common cold episodes in the previous year.

^c^ Adjusted for age, education, occupation, smoking status, BMI, physical activity, depressive symptoms, sleep duration, metabolic syndrome, calorie intake, vitamin C intake, and zinc intake.

^d^ Statistical significance: p < 0.05.

Similarly, Table 4 shows the ORs with 95% CI for having had common cold in the previous year based on the amount of alcohol intake. In comparison with the drinkers in the second tertile, the non-drinkers were significantly more likely to experience 2 or more episodes of common cold (OR, 1.843; 95% CI, 1.115–3.047), but the OR values for drinkers in the first and third tertiles were neither smaller nor larger. There was no association between the amount of alcohol and the occurrence of 1 or more episodes of common cold. Adjustment for potential confounders did not change the association between the amount of alcohol intake and the retrospective prevalence of common cold.

**Table 4:** Odds ratios for having had common cold in the previous year across alcohol intake amount

	**Amount of alcohol intake (g/day)**^**a**^			
	**Non-drinking (0)**	**1st (≤11.5)**	**2nd (11.5-35.8)**	**3rd (>35.8)**
≥1 common cold^b^ (cases/subjects)	91/149	141/250	136/249	130/251
Crude OR(95% CI)	1.304(0.862–1.971)	1.079 (0.755–1.530)	1	0.893 (0.628–1.269)
Adjusted OR^c^ (95% CI)	1.334(0.864–2.059)	0.950 (0.655–1.378)	1	0.939 (0.643–1.372)
≥2 common cold^b^ (cases/subjects)	38/149	51/250	39/249	51/251
Crude OR (95% CI)	1.843 (1.115–3.047) ^d^	1.380 (0.871–2.186)	1	1.373 (0.867–2.174)
Adjusted OR^c^ (95% CI)	2.155 (1.275–3.644) ^d^	1.317 (0.817–2.125)	1	1.387 (0.849–2.265)

^a^ Drinkers were categorized into tertiles.

^b^ Frequency of self-reported common cold episodes in the previous year.

^c^ Adjusted for age, education, occupation, smoking status, BMI, physical activity, depressive symptoms, sleep duration, metabolic syndrome, calorie intake, vitamin C intake, and zinc intake.

^d^ Statistical significance: p < 0.05.

## Discussion

The study revealed that alcohol intake frequency, not its amount, was associated with the retrospective prevalence of common cold. Moderate alcohol intake has been recognized as a behavior that effectively reduces health risks, such as the development of myocardial infarction [26] and all-cause mortality [27]. One of the previous studies showed that red wine intake reduced the risk of common cold, while intake of other beverages, such as beer and spirits, did not [7]. Give that the beneficial effect of red wine may be because of its higher antioxidant [28] and the anti-inflammatory capacity [29] of polyphenols, the difference seemed to be explained by significantly lower content of polyphenols in beer [30]. In the present study, however, the percentage of the subjects who drank wine was considerably lower than that of the subjects who drank beer or Japanese spirits. Because nearly two thirds of the drinkers consumed more than 2 types of beverages, mainly beer and Japanese spirits, and the numbers of subjects drinking each beverage alone were too small to analyze the independent effect of each type of alcoholic intake. Hence, although a possible effect of other components of beer and Japanese spirits cannot be excluded, alcohol itself may have a beneficial effect on reducing the incidence of common cold.

The associations between the retrospective prevalence and the amount and frequency of alcohol intake were stronger in subjects who had 2 or more episodes of common cold than those who experienced 1 or more episodes. Frequent incidence of common cold may be a reflection of higher susceptibility to common cold viruses, which, in turn, may be influenced by alcohol intake. Rhinovirus (RV) is one of the most common causative pathogens of common cold [31]. The replication of some types of RV, such as RV14 and RV16, is optimal at lower temperatures (33°C) and attenuated at higher temperatures (37°C) [32]. The average temperature in the upper respiratory tract is 32°C, as compared with 35°C in the sub-segmental bronchi [33]. Thus, airway temperature may be one of the key factors determining the susceptibility to RV infections, as well as the attacks of other temperature-sensitive viruses [34].

Raising airstream temperature is positively related with increasing blood flow of the mid-trachea [35]. Alcohol intake improves skin [36], cerebral [37] and coronary blood flow [38]. Hence, alcohol might increase airway blood flow and temperature. One of the mechanisms by which alcohol intake may increase airway temperature could be associated with nitric oxide (NO), a potent vasodilator [39] stimulated by ethanol [40-43]. Other factor which may be associated with the increased airway temperature is blood acetaldehyde level, which indirectly causes vasodilation [44] and increases after alcohol intake, resulting in increase blood flow [45]. The temporary effect of alcohol intake on increasing blood NO concentration is reported to last more than 1 hour and up to several hours [40,41], and blood acetaldehyde level returns to normal within 4 hours after alcohol intake [45]. Given that increased local blood flow in the upper respiratory tract contributes to protection against viruses, daily drinkers of small amounts of alcohol may have an advantage on viral protection over “weekend” or “occasional” drinkers.

There are possibilities that those who had history of stroke, cardiac disease, angina, or chronic renal failure may avoid or reduce alcohol drinking, and that those who have poorer health conditions may have higher incidence of common cold. In this study, however, only 1.7% of the participants had history of these diseases, and there were no skewed distribution of them across either the amount or the frequency of alcohol intake categories (data not shown). Moreover, in this study, there were no significant associations between the amount and frequency of alcohol intake and general health conditions such as physical activity level, depressive symptoms, and metabolic syndrome. Statistical adjustment for these variables did not affect the association between the retrospective prevalence of common cold and the frequency and amount of alcohol intake. Therefore, it is not likely that poorer health conditions have affected our results. Another possibility is that those who suffer common cold may have avoided drinking. Average incidence of common cold episodes per person in northern Japan is reported to be approximately 4 times per year among Japanese adults [46]. Sick days in a common cold episode commonly last for several days. It is therefore likely that the reduction in the amount or the frequency of alcohol during the sick days may not affect our results.

In an elderly population aged 66 to 79 years, those with depressive symptoms had higher incidence of common cold during a 1-year follow-up period [21]. In addition, previous studies showed that psychological stress was related with the risk of common cold [22]. However, in the current study, examining a slightly younger population, no association between depressive symptoms and retrospective common cold incidence was observed (data not shown); further, no association was found between alcohol intake and depressive symptoms.

A previous experimental study showed that those who sleep over 8 hours a day have lower risk of developing common cold [23]. A higher amount of alcohol intake per day has been associated with longer sleep duration [47]. Although the causality between drinking patterns and sleep duration cannot be completely explained, intake of alcohol as a sleep aid by the Japanese [48] may be one of its reasons. The association between sleep duration and common cold, however, was not observed in the current study. This may be due to the low number of subjects who slept 8 or more hours per day.

The present study has several limitations. First, the incidence of common cold was self-reported, without its specific diagnostic criteria and identification of viruses. Accordingly, there is a possibility that non-clinically diagnosed common cold was included. Second, recall bias may be involved, as the information was collected retrospectively. Patients with asthma [2] and COPD [3] are likely to develop common cold; consequently, those patients may tend to remember their common cold episodes better. However, these data are not available in the current study. Finally, obviously, excessive alcohol intake has negative effects on health and increases mortality [49,50]. Therefore, the results presented here should be interpreted with caution.

## Conclusion

The present study revealed that higher frequency of alcohol intake was associated with lower prevalence of self-reported common cold episodes in Japanese men. In contrast, the amount of alcohol intake was not associated with the retrospective prevalence of self-reported common cold. In order to determine whether a higher frequency of alcohol intake reduces the incidence of common cold, a long-term prospective study or randomized trials are required.

## Competing interests

The authors declare that they have no competing interests.

## Authors’ contributions

EO, KN, and RN designed the research, and interpreted the data; KN, YK, LG, HM, HG, MC, AO, YC, and RN participated in the acquisition of data; EO performed the statistical analysis, and writing the manuscript; KN, and RN supervised the analysis, and assisted in drafting and revising the manuscript. All authors read and approved the final manuscript.

## Pre-publication history

The pre-publication history for this paper can be accessed here:

http://www.biomedcentral.com/1471-2458/12/987/prepub

## References

[B1] Fendrick AM Monto AS Nightengale B Sarnes M The economic burden of non-influenza-related viral respiratory tract infection in the United States Arch Intern Med 2003 163 4 487 494 10.1001/archinte.163.4.487 12588210

[B2] Walter MJ Castro M Kunselman SJ Chinchilli VM Reno M Ramkumar TP Avila PC Boushey HA Ameredes BT Bleecker ER Predicting worsening asthma control following the common cold Eur Respir J 2008 32 6 1548 1554 10.1183/09031936.00026808 18768579 PMC2592508

[B3] Papi A Bellettato CM Braccioni F Romagnoli M Casolari P Caramori G Fabbri LM Johnston SL Infections and airway inflammation in chronic obstructive pulmonary disease severe exacerbations Am J Respir Crit Care Med 2006 173 10 1114 1121 10.1164/rccm.200506-859OC 16484677

[B4] Feeney A North F Head J Canner R Marmot M Socioeconomic and sex differentials in reason for sickness absence from the Whitehall II Study Occup Environ Med 1998 55 2 91 98 10.1136/oem.55.2.91 9614392 PMC1757555

[B5] Bramley TJ Lerner D Sames M Productivity losses related to the common cold J Occup Environ Med 2002 44 9 822 829 10.1097/00043764-200209000-00004 12227674

[B6] Cohen S Tyrrell DA Russell MA Jarvis MJ Smith AP Smoking, alcohol consumption, and susceptibility to the common cold Am J Public Health 1993 83 9 1277 1283 10.2105/AJPH.83.9.1277 8363004 PMC1694990

[B7] Takkouche B Regueira-Mendez C Garcia-Closas R Figueiras A Gestal-Otero JJ Hernan MA Intake of wine, beer, and spirits and the risk of clinical common cold Am J Epidemiol 2002 155 9 853 858 10.1093/aje/155.9.853 11978590

[B8] Global health observatory data repositoryhttp://app.who.int/ghodata 10.1080/02763869.2019.1693231 32069199

[B9] Statistical information: taxation of alcoholhttp://www.nta.go.jp/kohyo/tokei/kokuzeicho/sake2008/pdf/suryo.pdf

[B10] Guo H Niu K Monma H Kobayashi Y Guan L Sato M Minamishima D Nagatomi R Association of Japanese dietary pattern with serum adiponectin concentration in Japanese adult men Nutr Metab Cardiovasc Dis 2010 in press 10.1016/j.numecd.2010.06.006 20880683

[B11] Momma H Niu K Kobayashi Y Guan L Sato M Guo H Chujo M Otomo A Yufei C Tadaura H Skin advanced glycation end product accumulation and muscle strength among adult men Eur J Appl Physiol 2011 111 7 1545 1552 10.1007/s00421-010-1779-x 21188413 PMC3114099

[B12] Momma H Niu K Kobayashi Y Guan L Sato M Guo H Chujo M Otomo A Yufei C Tadaura H Skin advanced glycation end-product accumulation is negatively associated with calcaneal osteo-sono assessment index among non-diabetic adult Japanese men Osteoporos Int 2012 23 6 1673 1681 10.1007/s00198-011-1753-4 21901479 PMC3353116

[B13] Kobayashi Y Niu K Guan L Momma H Guo H Cui Y Nagatomi R Oral health behavior and metabolic syndrome and its components in adults J Dent Res 2012 91 5 479 484 10.1177/0022034512440707 22378694

[B14] Sasaki S Tanaka H Serum biomarker-based validation of a brief-type self-administered diet history questionnaire for Japanese subjects (in Japanese) A research for assessment of nutrition and dietary habit in “Kenko Nippon 21” 2005 The Study Group of Ministry of Health, Labor and Welfare of Japan, Tokyo 10 42

[B15] Tsugane S Fahey MT Sasaki S Baba S Alcohol consumption and all-cause and cancer mortality among middle-aged Japanese men: seven-year follow-up of the JPHC study Cohort I. Japan Public Health Center Am J Epidemiol 1999 150 11 1201 1207 10.1093/oxfordjournals.aje.a009946 10588080

[B16] Alberti KG Eckel RH Grundy SM Zimmet PZ Cleeman JI Donato KA Fruchart JC James WP Loria CM Smith SC Jr Harmonizing the metabolic syndrome: a joint interim statement of the International Diabetes Federation Task Force on Epidemiology and Prevention; National Heart, Lung, and Blood Institute; American Heart Association; World Heart Federation; International Atherosclerosis Society; and International Association for the Study of Obesity Circulation 2009 120 16 1640 1645 10.1161/CIRCULATIONAHA.109.192644 19805654

[B17] Craig CL Marshall AL Sjostrom M Bauman AE Booth ML Ainsworth BE Pratt M Ekelund U Yngve A Sallis JF International physical activity questionnaire: 12-country reliability and validity Med Sci Sports Exerc 2003 35 8 1381 1395 10.1249/01.MSS.0000078924.61453.FB 12900694

[B18] Fukuda K Kobayashi S A study on a self-rating depression scale Psychiatria et Neurologia Japonica 1973 75 673 679 4798819

[B19] Finklea JF Hasselblad V Sandifer SH Hammer DI Lowrimore GR Cigarette smoking and acute non-influenzal respiratory disease in military cadets Am J Epidemiol 1971 93 6 457 462 5562718 10.1093/oxfordjournals.aje.a121279

[B20] Matthews CE Ockene IS Freedson PS Rosal MC Merriam PA Hebert JR Moderate to vigorous physical activity and risk of upper-respiratory tract infection Med Sci Sports Exerc 2002 34 8 1242 1248 10.1097/00005768-200208000-00003 12165677

[B21] Kostka T Praczko K Interrelationship between physical activity, symptomatology of upper respiratory tract infections, and depression in elderly people Gerontology 2007 53 4 187 193 10.1159/000100017 17314485

[B22] Cohen S Tyrrell DA Smith AP Psychological stress and susceptibility to the common cold N Engl J Med 1991 325 9 606 612 10.1056/NEJM199108293250903 1713648

[B23] Cohen S Doyle WJ Alper CM Janicki-Deverts D Turner RB Sleep habits and susceptibility to the common cold Arch Intern Med 2009 169 1 62 67 10.1001/archinternmed.2008.505 19139325 PMC2629403

[B24] Van Straten M Josling P Preventing the common cold with a vitamin C supplement: a double-blind, placebo-controlled survey Adv Ther 2002 19 3 151 159 10.1007/BF02850271 12201356

[B25] Kurugol Z Akilli M Bayram N Koturoglu G The prophylactic and therapeutic effectiveness of zinc sulphate on common cold in children Acta Paediatr 2006 95 10 1175 1181 10.1080/08035250600603024 16982486

[B26] Mukamal KJ Conigrave KM Mittleman MA Camargo CA Stampfer MJ Willett WC Rimm EB Roles of drinking pattern and type of alcohol consumed in coronary heart disease in men N Engl J Med 2003 348 2 109 118 10.1056/NEJMoa022095 12519921

[B27] Paganini-Hill A Kawas CH Corrada MM Type of alcohol consumed, changes in intake over time and mortality: the Leisure World Cohort Study Age Ageing 2007 36 2 203 209 10.1093/ageing/afl184 17350977 PMC3377489

[B28] Maxwell S Cruickshank A Thorpe G Red wine and antioxidant activity in serum Lancet 1994 344 8916 193 194 7912786 10.1016/s0140-6736(94)92795-2

[B29] Estruch R Sacanella E Badia E Antunez E Nicolas JM Fernandez-Sola J Rotilio D de Gaetano G Rubin E Urbano-Marquez A Different effects of red wine and gin consumption on inflammatory biomarkers of atherosclerosis: a prospective randomized crossover trial. Effects of wine on inflammatory markers Atherosclerosis 2004 175 1 117 123 10.1016/j.atherosclerosis.2004.03.006 15186955

[B30] Gorinstein S Caspi A Zemser M S T Comparative contents of some phenolics in beer, red and white wines Nutr Res 2000 20 1 131 139 10.1016/S0271-5317(99)00145-1

[B31] Makela MJ Puhakka T Ruuskanen O Leinonen M Saikku P Kimpimaki M Blomqvist S Hyypia T Arstila P Viruses and bacteria in the etiology of the common cold J Clin Microbiol 1998 36 2 539 542 9466772 10.1128/jcm.36.2.539-542.1998 PMC104573

[B32] Papadopoulos NG Sanderson G Hunter J Johnston SL Rhinoviruses replicate effectively at lower airway temperatures J Med Virol 1999 58 1 100 104 10.1002/(SICI)1096-9071(199905)58:1<100::AID-JMV16>3.0.CO;2-D 10223554

[B33] McFadden ER Jr Pichurko BM Bowman HF Ingenito E Burns S Dowling N Solway J Thermal mapping of the airways in humans J Appl Physiol 1985 58 2 564 570 3980358 10.1152/jappl.1985.58.2.564

[B34] du Prel JB Puppe W Grondahl B Knuf M Weigl JA Schaaff F Schmitt HJ Are meteorological parameters associated with acute respiratory tract infections? Clin Infect Dis 2009 49 6 861 868 10.1086/605435 19663691

[B35] Gilbert I Regnard J Lenner K Nelson J McFadden EJ Intrathoracic airstream temperatures during acute expansions of thoracic blood volume Clin Sci 1999 81 655 661 1661652 10.1042/cs0810655

[B36] Iwase S Matsukawa T Ishihara S Tanaka A Tanabe K Danbara A Matsuo M Sugiyama Y Mano T Effect of oral ethanol intake on muscle sympathetic nerve activity and cardiovascular functions in humans J Auton Nerv Syst 1995 54 3 206 214 10.1016/0165-1838(95)00022-P 7490422

[B37] Christie IC Price J Edwards L Muldoon M Meltzer CC Jennings JR Alcohol consumption and cerebral blood flow among older adults Alcohol 2008 42 4 269 275 10.1016/j.alcohol.2008.03.132 18539247 PMC2527510

[B38] Kiviniemi TO Saraste A Toikka JO Saraste M Raitakari OT Parkka JP Lehtimaki T Hartiala JJ Viikari J Koskenvuo JW A moderate dose of red wine, but not de-alcoholized red wine increases coronary flow reserve Atherosclerosis 2007 195 2 e176 181 10.1016/j.atherosclerosis.2007.06.004 17662293

[B39] Palmer RM Ferrige AG Moncada S Nitric oxide release accounts for the biological activity of endothelium-derived relaxing factor Nature 1987 327 6122 524 526 10.1038/327524a0 3495737

[B40] Matsuo S Nakamura Y Takahashi M Ouchi Y Hosoda K Nozawa M Kinoshita M Effect of red wine and ethanol on production of nitric oxide in healthy subjects Am J Cardiol 2001 87 8 1029 1031 A1026 10.1016/S0002-9149(01)01446-1 11306004

[B41] Oekonomaki E Notas G Mouzas IA Valatas V Skordilis P Xidakis C Kouroumalis EA Binge drinking and nitric oxide metabolites in chronic liver disease Alcohol Alcohol 2004 39 2 106 109 14998825 10.1093/alcalc/agh030

[B42] Abou-Agag LH Khoo NK Binsack R White CR Darley-Usmar V Grenett HE Booyse FM Digerness SB Zhou F Parks DA Evidence of cardiovascular protection by moderate alcohol: role of nitric oxide Free Radic Biol Med 2005 39 4 540 548 10.1016/j.freeradbiomed.2005.04.007 16043025

[B43] Husain K Mejia J Lalla J Kazim S Dose response of alcohol-induced changes in BP, nitric oxide and antioxidants in rat plasma Pharmacol Res 2005 51 4 337 343 10.1016/j.phrs.2004.10.005 15683747

[B44] Eriksson CJ The role of acetaldehyde in the actions of alcohol (update 2000) Alcohol Clin Exp Res 2001 5 Suppl ISBRA 15S 32S 11391045 10.1097/00000374-200105051-00005

[B45] Mizoi Y Tatsuno Y Adachi J Kogame M Fukunaga T Fujiwara S Hishida S Ijiri I Alcohol sensitivity related to polymorphism of alcohol-metabolizing enzymes in Japanese Pharmacol Biochem Behav 1983 18 Suppl 1 127 133 6356156 10.1016/0091-3057(83)90159-4

[B46] Satomura K Kitamura T Kawamura T Shimbo T Watanabe M Kamei M Takano Y Tamakoshi A Investigators-I GC Prevention of upper respiratory tract infections by gargling: a randomized trial Am J Prev Med 2005 29 4 302 307 10.1016/j.amepre.2005.06.013 16242593

[B47] Gottlieb DJ Redline S Nieto FJ Baldwin CM Newman AB Resnick HE Punjabi NM Association of usual sleep duration with hypertension: the Sleep Heart Health Study Sleep 2006 29 8 1009 1014 16944668 10.1093/sleep/29.8.1009

[B48] Kaneita Y Uchiyama M Takemura S Yokoyama E Miyake T Harano S Asai T Tsutsui T Kaneko A Nakamura H Use of alcohol and hypnotic medication as aids to sleep among the Japanese general population Sleep Med 2007 8 7–8 723 732 17512790 10.1016/j.sleep.2006.10.009

[B49] Breslow RA Graubard BI Prospective study of alcohol consumption in the United States: quantity, frequency, and cause-specific mortality Alcohol Clin Exp Res 2008 32 3 513 521 10.1111/j.1530-0277.2007.00595.x 18215212

[B50] Sadakane A Gotoh T Ishikawa S Nakamura Y Kayaba K Amount and frequency of alcohol consumption and all-cause mortality in a Japanese population: the JMS Cohort Study J Epidemiol 2009 19 3 107 115 10.2188/jea.JE20081003 19398849 PMC3924134

